# Hyaladherins May be Implicated in Alcohol-Induced Susceptibility to Bacterial Pneumonia

**DOI:** 10.3389/fimmu.2022.865522

**Published:** 2022-05-12

**Authors:** Kathryn M. Crotty, Samantha M. Yeligar

**Affiliations:** ^1^Department of Medicine, Division of Pulmonary, Allergy, Critical Care and Sleep Medicine, Emory University, Atlanta, GA, United States; ^2^Atlanta Veterans Affairs Health Care System, Decatur, GA, United States

**Keywords:** hyaluronan, alcohol use disorder, pneumonia, hyaladherin, immunity

## Abstract

Although the epidemiology of bacterial pneumonia and excessive alcohol use is well established, the mechanisms by which alcohol induces risk of pneumonia are less clear. Patterns of alcohol misuse, termed alcohol use disorders (AUD), affect about 15 million people in the United States. Compared to otherwise healthy individuals, AUD increase the risk of respiratory infections and acute respiratory distress syndrome (ARDS) by 2-4-fold. Levels and fragmentation of hyaluronic acid (HA), an extracellular glycosaminoglycan of variable molecular weight, are increased in chronic respiratory diseases, including ARDS. HA is largely involved in immune-assisted wound repair and cell migration. Levels of fragmented, low molecular weight HA are increased during inflammation and decrease concomitant with leukocyte levels following injury. In chronic respiratory diseases, levels of fragmented HA and leukocytes remain elevated, inflammation persists, and respiratory infections are not cleared efficiently, suggesting a possible pathological mechanism for prolonged bacterial pneumonia. However, the role of HA in alcohol-induced immune dysfunction is largely unknown. This mini literature review provides insights into understanding the role of HA signaling in host immune defense following excessive alcohol use. Potential therapeutic strategies to mitigate alcohol-induced immune suppression in bacterial pneumonia and HA dysregulation are also discussed.

## Introduction

Excessive alcohol use associated with alcohol use disorders (AUD) (1) is linked to over 5 million annual deaths globally (2), in part due to an increased risk of respiratory infections (3) and acute respiratory distress syndrome (ARDS) (4). Pneumonia is a serious respiratory infection that is caused by at least one of several opportunistic bacteria, viruses, or fungi. Nearly 44,000 people die annually due to pneumonia in the United States, while another 1.5 million are hospitalized for pneumonia as a primary diagnosis (5). Ethanol (EtOH) impairs mucociliary clearance in the upper airway (6, 7) and diminishes innate immune defense in the lower airway by impairing the ability of alveolar macrophages (AM) to phagocytose pathogens (8–11), such as bacterial pneumonia (11, 12). Upon pneumonia-associated microbial evasion of host immune defense mechanisms in the upper airway, microbial culture in the lower airways causes pneumonia. This mini review focuses on molecular mechanisms, such as that of hyaluronic acid (HA), that may be implicated in increased susceptibility to bacterial pneumonia during acute and chronic EtOH use. Modulation of HA metabolism, signaling, and intracellular communication that impact cellular immune functions during bacterial pneumonia may pave the way for future investigations on how alterations in the extracellular matrix may be exacerbated by excessive alcohol use.

## Extracellular Matrix in the Lung

The extracellular matrix is a dynamic environment, rich with proteins, carbohydrates, and other significant structural molecules. In diseased states, additional matrix deposition results in diminished intracellular communication and progression to fibrosis. AUD-associated risk of pneumonia and ARDS (3, 4) precedes pulmonary fibrosis and loss of function if unresolved (13).

Hyaluronic acid (HA), an extracellular matrix glycosaminoglycan, is essential for maintaining tissue structure, promoting cell survival, and regulating inflammation and leukocyte motility after pulmonary injury (14–19). Further, accumulation of HA fragments is associated with chronic pulmonary inflammation mediated by innate immune cells (20–27). Increased HA synthesis and fragmentation is commonly involved in pulmonary disease pathology including fibrotic diseases (27–30), excessive remodeling (14, 18, 31, 32), and inflammation (15, 24, 33–37). In non-pathologic conditions, HA is expressed at very low concentrations in bronchoalveolar lavage fluid (38, 39) but is increased during pulmonary inflammation and pneumonia infections from *Klebsiella pneumoniae (* 40*)* and *Escherichia coli* (41, 42).

Bacterial pneumonia clearance depends on dynamic, but regulated, HA metabolism and HA binding protein signaling (36, 40–44). Regulation of HA size and signaling through cell surface immune receptors is necessary to mobilize leukocytes, including alveolar macrophages, for recognition and destruction of infectious pathogens in those with AUD. Remodeling after respiratory infections is crucial and involves a restoration of HA dynamics coinciding with decreases in bacterial colonization, inflammation, and leukocyte recruitment.

## HA Signaling: Hyaladherins and HA-Protein Interactions

Hyaladherins are HA binding proteins that transmit changes in the extracellular matrix to cell signals for altered intra- or inter-immune cell function (14) through intermediate proteoglycans (45, 46) or by ionic HA binding to membrane proteins (47, 48). Although alcohol diminishes the ability of alveolar macrophages to recognize and clear pathogens, the role of HA on bacterial recognition during excessive alcohol use is largely unknown.

### CD44 and CHI3L1

Cluster of differentiation 44 (CD44) is a hyaladherin that spans the cellular membrane, binds HA, and internalizes HA for lysosomal degradation by hyaluronidase enzymes (49, 50). CD44 is the primary cell surface receptor for HA binding in lymphocytes (51–53) and forms an anti-apoptotic coat of HA around alveolar macrophages (54). Therefore, CD44 is crucial for HA metabolism and signaling in leukocytes. Granulocyte-macrophage colony stimulating factor (GM-CSF) and peroxisome proliferator-activated receptor gamma (PPARγ) agonism induce expression of CD44 in monocytes that do not readily bind HA (54). However, chronic alcohol diminishes GM-CSF and PPARγ (11, 55) in primary alveolar macrophages, potentially decreasing their ability to form an anti-apoptotic HA coat for signaling with other hyaladherins.

Patients with eosinophilic pneumonia have high concentrations of CD44, HA, and interleukin-5 in their bronchoalveolar fluid. In contrast, CD44 deficient mice show decreased HA content after *Streptococcus pneumoniae* but increased HA in response to *E. coli* infection (41), suggesting that bacterial strains differentially influence host HA matrices. Yet, these studies do not address altered HA binding or signaling as mechanisms for worsened bacterial pneumonia. While altered CD44 expression following alcohol use may be one mechanism of bacterial pneumonia pathogenesis, altered HA molecular weight or indirect HA signaling may also impact inflammatory signaling and the innate immune response in leukocytes.

For indirect immune cell signaling, chitinase-3 like-protein-1 (CHI3L1) forms an intermediate bond between CD44 and HA (56). Through HA binding to CHI3L1 (57, 58), lysosomal degradation of HA by CD44 internalization is inhibited. Thus, CHI3L1 indirectly inhibits HA uptake and degradation through CD44 mediated internalization, suggesting CHI3L1 as an important regulator of HA metabolism. CHI3L1 is expressed in macrophages, neutrophils and endothelial cells and is necessary for antigen response, oxidant injury response, inflammation, and macrophage phenotype in the lung (59). Alcohol and high CHI3L1 levels have been linked to the progression of liver injury and fibrosis (60–62), but not yet in alcohol and bacterial pneumonia.

In bacterial pneumonia, CHI3L1 activity promotes innate immune defenses by sensing oxidant stress, cytokines, growth factors and miRNAs in the extracellular environment. Patients hospitalized with pneumonia have increased levels of CHI3L1 in serum (44, 63, 64). Additionally, *S. pneumoniae* induces CHI3L1 expression, but mice lacking CHI3L1 have reduced bacterial clearance and enhanced mortality following *S. pneumoniae* infection (43). These studies suggest CD44 and CHI3L1 as important regulators of innate immunity in the lung during bacterial pneumonia. Further, these studies provide CD44 and CHI3L1 as targetable mechanisms for treating bacterial pneumonia in those with AUD.

### HA Heavy Chain Formation

Tumor necrosis factor-stimulated gene-6 (TSG-6) is secreted by immune cells (65) and catalyzes inter-α-trypsin-inhibitor (IαI)-heavy chain complex to HA through pentatraxin 3 (PTX3) (66). Together, these molecular components generate a heavy chain HA matrix involved in airway inflammation (67), hyperresponsiveness (68–71) and toll-like receptor 4 (TLR4)-mediated lung injury (35, 69), possibly through PTX3 stimulation by TLR signaling (72). IαI attenuates lung injury in a porcine model of lipopolysaccharide (LPS)-induced sepsis (73), and PTX3 deficiency worsens LPS-induced lung injury. TSG-6 expression in cultured U-937 monocytes is enhanced by *Staphylococcus aureus* and *Chlamydia pneumoniae (* 74*)*, suggesting enhanced expression in some strains of bacterial pneumonia. Further, PTX3 is involved in microbial recognition and innate immunity through recruitment of leukocytes and binding to *K. pneumoniae, Pseudomonas aeruginosa, Salmonella enterica, S. aureus, Neisseria meningitidis*, and *S. pneumoniae (* 75–77*)*. Altogether, there is sufficient evidence for the role of heavy chain HA matrices in bacterial pneumonia, but further studies are needed to elucidate if PTX3 involvement in heavy chain HA formation is due to production by host or pathogen.

Little is known about heavy chain HA formation during excessive alcohol use. If heavy chain HA formation is involved in lung injury amelioration during bacterial pneumonia, disruptions in this process may lead to further lung injury and possibly sepsis. The risk of developing sepsis from pneumonia increases from 35% to 60% in people with AUD (4). EtOH feeding to C57BL/6 mice significantly diminished survival rates and lung PTX3 expression in a model of sepsis, and delayed tumor necrosis factor α (TNFα) level increases in plasma (78). Similarly, in a binge drinking mouse model of gram-negative bacterial lung infection, plasma TNFα was suppressed even while bacterial colonization was increased (79). Overall, these studies suggest that sepsis after excessive alcohol use not due to lack of inflammatory TNFα signaling. Rather, alterations in PTX3 disrupt HA heavy matrix formation and may be a mechanism for deranged immune function in those with AUD.

### Versican and TLRs

Lecticans are HA-binding proteoglycans, containing chondroitin sulfate side chains, that ionically bind to HA through clusters of positively charged amino acids forming the link domain (48, 53). Little is known about how lecticans are impacted in bacterial pneumonia; however, levels of hyaluronan and the lectican, versican, increase during lung injury (38, 80, 81), perhaps by HA synthase regulation (82, 83). Although rats exposed to fetal alcohol showed a decrease in synaptic versican (84), the role of versican in alcohol-induced lung derangements continue to be an active area of investigation.

TLRs bind to hyaladherins and are known mediators of the inflammatory response during bacterial pneumonia. Like HA, versican can act as a danger associated molecular pattern for TLR signaling in alveolar macrophages (85, 86). Versican is augmented in the lungs of adult mice exposed to *P. aeruginosa* and upon TLR agonism (87). Comparatively, conditional versican deficiency in myeloid cells reduced inflammatory cell recruitment to the lungs (88). LPS stimulation of the TLR4/Trif pathway increases HA and versican levels in bone marrow derived macrophages *in vitro* and in murine alveolar macrophages (42, 88), but there is a lack of similar studies with gram positive bacteria.

Defects in TLR signaling predispose an individual to immunodeficiency that can result in severe bacterial pneumonia (89). Further, the versican receptors TLR2 and TLR4 are affected by excessive alcohol use. TLR2 and TLR4 do not bind HA but have been hypothesized to interact with HA through clustering of other matrix or membrane proteins and proteoglycans, like versican. Individuals with alcohol use disorders showed significant increases in TLR2; those with AUD and cannabis use exhibited significant increases in TLR6 (90). No experimental groups had increased TLR4 expression in that study, but another study showed that alcohol exposure induced TLR4 endocytosis in alveolar macrophages, limiting TLR4 activity for the recognition of pathogens (11). These results suggest that TLR expression or signaling may compensate for impaired bacterial recognition in those who have an AUD and bacterial pneumonia. Other membrane hyaladherins can also bind HA simultaneously to influence leukocyte phenotype (91) and affect pro- or anti-inflammatory signaling depending on the binding protein. While it is not known if hyaluronan or any binding partners interact with the other TLRs, these studies identified multiple targets for therapeutic intervention.

### RHAMM, HABP1 and HABP2

Receptor for HA mediated motility (RHAMM), and HA binding protein 1 and 2 (HABP1, HABP2) are expressed ubiquitously and have multiple binding partners, including HA (92, 93). RHAMM contains putative binding domains for HA (94), but RHAMM is mainly expressed intracellularly (93, 95–97) to participate in signaling excluding HA. However, it is possible that HA binds to hyaladherins within the cell membrane because several hyaladherins are expressed intracellularly. Upon HA interaction with RHAMM, cell migration is promoted, influencing tissue remodeling or immune cell trafficking (98). In mice, there is increased membrane expression of RHAMM following lung injury (99). Further, RHAMM can compensate for CD44 through increased HA binding without increased RHAMM expression, indicating convergence of HA signaling pathways (100).

RHAMM is implicated in acute lung injury (101), and alcohol use exacerbates acute lung injury (4, 8, 13, 102, 103). However, it is not yet known how alcohol consumption directly affects RHAMM in any organ system. Past work has shown that RHAMM and transforming growth factor beta (TGFβ) work collectively to promote cell motility (104). Alcohol use inhibits inflammatory cytokines while stimulating TGFβ, which acts as an inhibitory cytokine in human monocytes exposed to bacterial stimuli (105). In contrast, some studies show that alcohol induces lung injury through proinflammatory pathways and promote fibrosis by stimulating TGFβ1 activity (106, 107). In alveolar macrophages, alcohol-induced oxidative stress through TGFβ1 regulation of NADPH oxidases diminished alveolar macrophage function (108). Altogether, TGFβ1 is clearly involved in immune dysfunction following alcohol use, but more information is necessary to conclude that changes in TGFβ1 contribute to alterations in RHAMM signaling.

HABP1, also known as p32 or gClqR, can be found at the cell surface with higher affinity for HA corresponding to ionic strength and acidic environments (109), and HA binding to HABP1 can inhibit HA degradation by *S. pneumoniae* hyaluronidases (110). Bacteria express hyaluronidase proteins that degrade host HA matrices to allow for greater bacterial movement; thus, HABP1 activity is an endogenous antibacterial host defense. In humans, HABP1 assists in the regulation of HA metabolism in non-diseased states. While there is little known about HABP1 involvement in bacterial pneumonia, HABP1 activity is well described in cancer and mitochondrial biology. Alcohol exposure impairs alveolar macrophage ability to phagocytose pathogens (8–11) via increased cellular oxidative stress (111), mitochondrial redox imbalance (112, 113), and impaired mitochondrial bioenergetics (114). Mitochondrial HABP1 regulates oxidative phosphorylation (115, 116) by maintaining mitochondrial protein translation (117), and cleavage of HABP1 by caspase-1 shifts cancer cell phenotype toward glycolysis (118). In human lung cancers, HABP1 is highly expressed, leading to altered nuclear factor kappa B (NFκB) activity and cell proliferation (119), revealing a role for HABP1 in the lung microenvironment.

HABP2, also known as factor VII activating protease or plasma hyaluronan binding protein, is extracellular. High molecular weight HA inhibits HABP2’s activity to maintain barrier integrity while low molecular weight HA prevents a leaky barrier (120, 121). Normal barrier function prevents bacterial spread into the vasculature during bacterial pneumonia that would otherwise result in sepsis. Further, alcohol impairs pulmonary barrier function (122, 123). In the lung, HABP2 may be involved in LPS-induced lung injury (121) and ARDS (124) primarily through its role in modulating lung barrier integrity. In patients with ARDS, HABP2 levels and activity are increased in alveolar macrophage, epithelial, and endothelial cells (124), and chronic alcohol use elevates the risk for ARDS (4).

*In vivo* HABP2 silencing by small interfering RNA attenuated LPS-mediated lung injury and hyperpermeability, indicating a possible therapeutic strategy for bacterial pneumonia in those with AUD-induced barrier dysfunction. Additionally, HABP2 primarily binds to cell surface protease-activated receptors (PAR) (125), and silencing of PAR1 and PAR3 can attenuate LPS-mediated barrier dysfunction (121). Mice with PAR2 genetic deletions exhibited severe lung inflammation, neutrophil accumulation, and diminished macrophage and neutrophil bacterial phagocytosis in a model of *P. aeruginosa*. These alterations were attenuated by PAR2 activation (126), indicating a possible role for HABP2 in bacterial pneumonia clearance. Other studies show similar roles for PARs in bacterial pneumonia pathology (126–128); however, this mechanism needs to be further elucidated since HABP1 and the PARs each have multiple binding partners.

## Discussion

**Figure 1:**
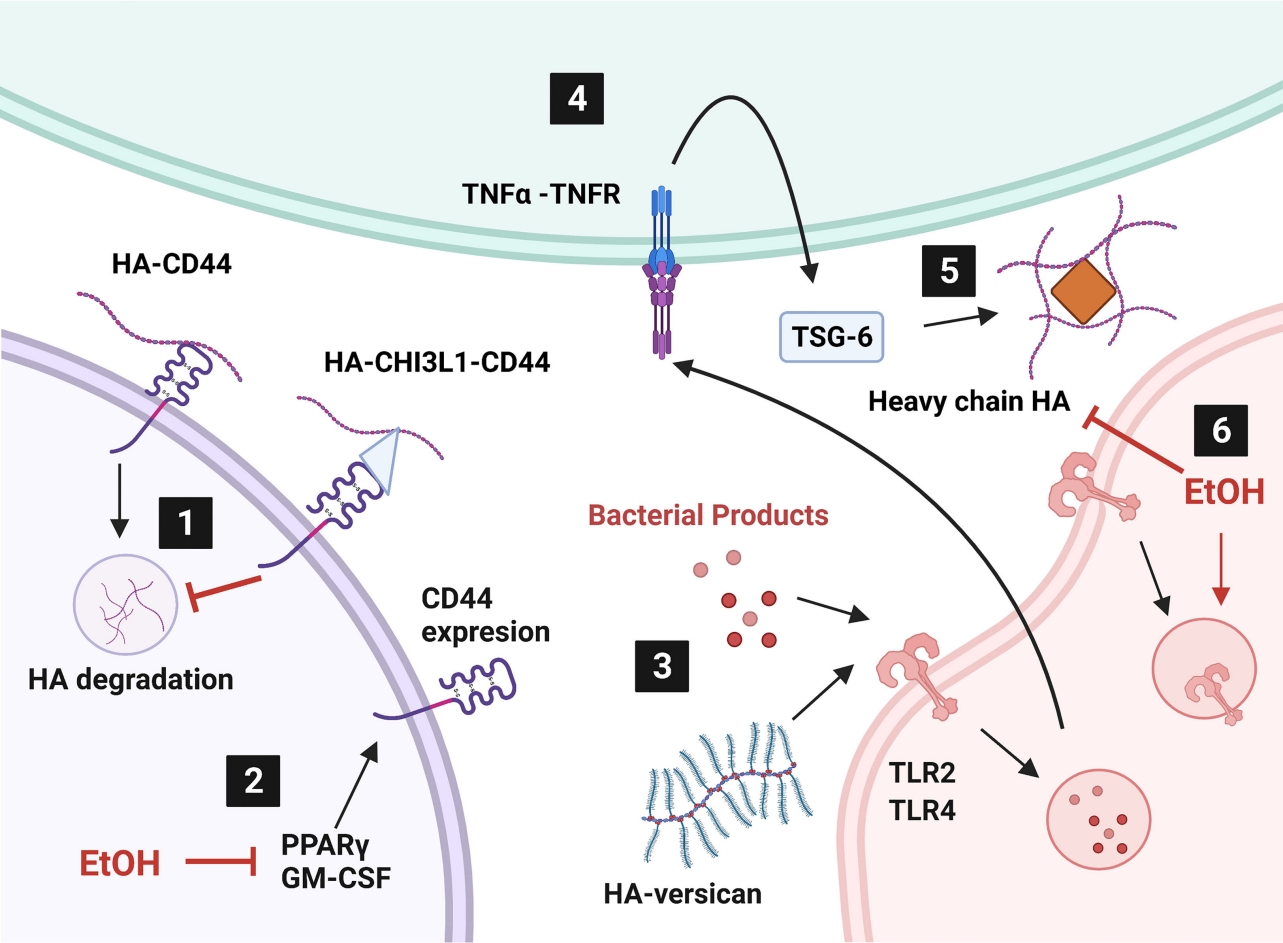
Alcohol affects hyaladherin signaling in the lung. 1) Internalization and degradation of hyaluronic acid (HA) is inhibited by overproduction of chitinase-3 like-protein-1 (CHI3L1). 2) Ethanol (EtOH) diminishes peroxisome proliferator activated receptor gamma (PPARγ) and granulocyte-macrophage colony stimulating factor (GM-CSF) levels. 3) HA-versican competes with bacterial products for toll-like receptor (TLR) signaling. 4) TLR signaling induces tumor necrosis factor alpha (TNFα) production. TNFα stimulates TNFα-stimulated gene-6 (TSG-6) expression. 5) TSG-6 catalyzes heavy chain HA matrix formation through pentatraxin 3 (PTX3, orange diamond). 6) EtOH induces TLR4 internalization and heavy chain formation by decreasing TNFα. Created with BioRender.com.

This mini review addresses modulation of HA signaling by alcohol and bacterial pneumonia. CD44 and RHAMM are involved in HA metabolism, signaling, and intracellular communication. CHI3L1, IαI, TSG-6, PTX3, and versican all act as intermediates between HA and membrane signaling proteins, like CD44 and TLRs. Herein we also review how HA modulates cellular energy metabolism through HABP2 and intracellular signaling. Another hyaladherin, lymphatic vessel endothelial cell receptor 1 (LYVE-1), binds HA for immune cell motility and HA metabolism but was not discussed in detail due to its low expression in the lungs. Nevertheless, CD44 and LYVE-1 jointly assist in immune cell migration within the lymphatic system (129–131) to traffic cells to the lungs during bacterial pneumonia. HA-hyaladherin interactions additionally assist with leukocyte motility. In summary, changes in the extracellular matrix impact cellular signaling in bacterial pneumonia that can be exacerbated by excessive alcohol use but there is much to learn still. Nevertheless, targeting hyaladherins may be a potential therapeutic strategy for mitigating lung injury in those with alcohol use disorders. These pathways have been summarized in **Figure 1**.

### Controversies in the HA Field

Is increased HA production during lung disease pathological and does it need to be “fixed?” HA concentration increases, but average molecular weight decreases, in multiple pulmonary diseases involving immune dysfunction and inflammation. However, the mechanisms of HA signaling based on variations in molecular weight remain controversial in the field. Increased HA production appears to decrease leukocyte mobility and bacterial spread in pneumonia due to higher viscosity. However, increased HA production may aid in leukocyte motility through endogenous hyaladherins while preventing bacterial spread because of their lack of the same receptors.

Further, fragmented HA is thought to be pro-inflammatory while endogenous high molecular weight HA is anti-inflammatory (25, 34, 132). It is also clear that bacteria contain hyaluronidases to degrade host HA matrices, and fragmented HA can act as a danger associated molecular pattern for immune cell release of key immune factors. Our group has hypothesized that alcohol increases high molecular weight HA synthesis, thereby decreasing necessary pro-inflammatory signaling from fragmented HA. However, size classifications remain controversial in the field since “fragmented HA” or “low molecular weight HA” could range from HA chains of a few polysaccharides to 500 kD. Future studies should be done to clarify the immune response of leukocytes to different sized HA polymers to confirm past results.

### Therapeutic Potential

Although the risk AUD individuals for getting sepsis and ARDS from pneumonia is approximately double that of non-AUD individuals (4), treatment strategies are comparable between AUD and non-AUD individuals. There are several FDA approved modulators of HA or HA binding proteins that are available by prescription or as a clinical treatment; however, additional studies on HA modulation in bacterial pneumonia and alcohol are needed before therapeutic targeting of these pathways in people with AUD can take place. Targeting bacterial protein influence in host HA matrices and barrier dysfunction go hand-in-hand. As bacteria spread and host lung cell apoptosis persists, cellular barriers are broken down. Use of current small molecule inhibitors of bacterial hyaluronidases are insufficient as a therapeutic strategy because they have low specificity and potency. Bacteria contain some hyaluronidases that are different than those in humans. Therefore, upregulation of host defenses against bacterial hyaluronidases, like HABP1, may work as an alternative treatment to prevent uncontrolled bacterial proliferation.

Proposed mechanisms of EtOH-induced oxidative stress in alveolar macrophage include loss of PPARγ activity (8, 11, 111), which is diminished following alcohol exposure (11, 55, 111). Rosiglitazone and pioglitazone, PPARγ agonists, improve EtOH-induced alveolar macrophage oxidative stress (9), mitochondrial-derived ROS (114**)**, and dysfunctional phagocytosis and clearance of *K. pneumoniae (* 11). Further, pioglitazone, reversed alcohol-induced derangements phagocytosis in alveolar macrophages (11, 55, 111). Because mitochondrial derived ATP is necessary for high energy processes, like phagocytosis, impaired mitochondrial function is one explanation for why alcohol impairs alveolar macrophage phagocytic ability. Identifying alcohol-induced mechanisms that impair HA signaling could further elucidate underlying mitochondrial dysfunction in alveolar macrophages.

In conclusion, AUDs increase the risk of respiratory infections and levels of the extracellular matrix component, HA, are increased in chronic respiratory diseases. HA signaling through hyaladherins are affected by alcohol use, which could modify inflammation and immune cell activity during bacterial pneumonia. The role of hyaladherins in alcohol-induced immune dysfunction is still largely unknown. This mini review highlights the necessity for future studies to provide insight into understanding the role of HA and its binding partners in host immune defense following excessive alcohol use.

## Author Contributions

KMC outlined and prepared the manuscript; SMY outlined and prepared the manuscript. All authors contributed to the article and approved the submitted version.

## Funding

This work was supported in part by grants from: the National Institute on Alcohol Abuse and Alcoholism (F31AA029938) to KMC (ORCID ID: 0000-0002-9461-4032) and (R01AA026086) to SMY (ORCID ID: 0000-0001-9309-0233) as well as the National Institute of General Medical Sciences (T32GM008602) to Randy A. Hall. The contents of this report do not represent the views of the Department of Veterans Affairs or the US Government.

## Conflict of Interest

The authors declare that the research was conducted in the absence of any commercial or financial relationships that could be construed as a potential conflict of interest.

## Publisher’s Note

All claims expressed in this article are solely those of the authors and do not necessarily represent those of their affiliated organizations, or those of the publisher, the editors and the reviewers. Any product that may be evaluated in this article, or claim that may be made by its manufacturer, is not guaranteed or endorsed by the publisher.

## Abbreviations

AUD, alcohol use disorder; ARDS, acute respiratory distress syndrome; HA, hyaluronic acid; EtOH, ethanol; CD44, Cluster of differentiation 44; GM-CSF, Granulocyte-macrophage colony stimulating factor; PPARγ, peroxisome proliferator activating receptor gamma; CHI3L1, chitinase-3 like-protein-1; TSG-6, Tumor necrosis factor-stimulated gene-6; PTX3, pentatraxin 3; TLR, Toll-like receptor; IαI, inter-α-trypsin-inhibitor; LPS, lipopolysaccharide; TNFα, tumor necrosis factor α; RHAMM, Receptor for HA mediated motility; HABP, hyaluronic acid binding protein; TGFβ, transforming growth factor beta; NFκB, nuclear factor kappa B; PAR, protease-activated receptors; LYVE-1, lymphatic vessel endothelial cell receptor 1.
